# Determination of chemical ordering in the complex perovskite Pb(Cd_1/3_Nb_2/3_)O_3_

**DOI:** 10.1107/S2052252518013805

**Published:** 2018-10-24

**Authors:** Caiyan Wang, Zhengqian Fu, Nan Zhang, Marek Paściak, Jian Zhuang, Zenghui Liu, Wei Ren, Zuo-guang Ye

**Affiliations:** a Electronic Materials Research Laboratory, Key Laboratory of the Ministry of Education and International Center for Dielectric Research, School of Electronic and Information Engineering, Xi’an Jiaotong University, Xi’an 710049, People’s Republic of China; b Analysis and Testing Center for Inorganic Materials, Shanghai Institute of Ceramics, Chinese Academy of Sciences, Shanghai 200050, People’s Republic of China; c Institute of Physics, The Academy of Sciences of the Czech Republic, Na Slovance 2, Prague 182 21, Czech Republic; d Department of Chemistry and 4D LABS, Simon Fraser University, Burnaby, British Columbia V5A 1S6, Canada

**Keywords:** chemical ordering, diffuse scattering, electron microscopy, atomic resolution EDS, perovskites, dielectric permittivity, relaxors

## Abstract

Pure-phase Pb(Cd_1/3_Nb_2/3_)O_3_ (PCN) single crystals and ceramics with a complex perovskite structure are synthesized for the first time. The local chemical ordering in PCN has been investigated by X-ray diffraction (including diffuse scattering) and Cs-corrected transmission electron microscopy experiments. It is concluded that the PCN samples have large coherent chemical ordering regions that even extend to the long range, and the ordering model is consistent with β-type chemical ordered regions. The antiphase domain boundaries were also observed. Two dielectric anomaly peaks were found in these two types of samples, one of which indicates possible relaxor behaviour. The novel structure of the completely ordered regions and its relationship with the electrical properties make PCN a unique material for the fundamental understanding of chemically substituted perovskites.

## Abstract 2

Using various experimental methods including X-ray diffraction and electron microscopy, this work determines the range and nature of the chemical ordering in Pb(Cd_1/3_Nb_2/3_)O_3_ (PCN). The influence of the chemical ordering on the dielectric property is also discussed, based on the similarities and differences between PCN and other classical relaxors.

## 1. Introduction   

Lead-based perovskite materials such as Pb(Mg_1/3_Nb_2/3_)O_3_ (PMN), Pb(Zn_1/3_Nb_2/3_)O3 (PZN), Pb(Sc_1/2_Nb_1/2_)O_3_ (PSN) and Pb(Sc_1/2_Ta_1/2_)O_3_ (PST) have been the subject of extensive research during recent years because of their unique relaxor behaviour (Bokov & Ye, 2006 ▸; Hirota *et al.*, 2002 ▸; Gehring *et al.*, 2001 ▸; Stenger & Burggraaf, 1980*a* ▸,*b* ▸; Setter & Cross, 1980*b* ▸). In contrast to classical ferroelectrics, in which a sharp change of dielectric permittivity occurs at the phase transition from polar to nonpolar states, relaxors usually have a significantly diffuse dielectric peak over a wide temperature range and also have a broad range of dependence on the frequency dispersion. It is believed that relaxor behaviour is related to the partially ordered distributions of different ions on equivalent crystallographic sites, which could lead to compositional disorder and polar inhomogeneity (so-called polar nanoregions, PNRs) at the atomic scale. The dynamics and the sizes of the PNRs and the existence of compositionally ordered regions (CORs) are commonly believed to give rise to relaxor behaviour (Ye *et al.*, 2003 ▸; Kalinin *et al.*, 2010 ▸; Bokov *et al.*, 2011 ▸). However, a more definitive picture of a nanoscale structural description of relaxors is still lacking, and the direct relationship between local disorder and relaxor behaviour is not yet well understood.

Many Pb-based relaxors are complex perovskites, with two or more types of elements occupying the B site, which leads to a partial B-site cation-ordered structure (Randall & Bhalla, 1990 ▸; Burton *et al.*, 2005 ▸; Zhao *et al.*, 2009 ▸). PMN and PZN are among the most studied Pb(B′_1/3_B′′_2/3_)O_3_-type relaxor compounds that exhibit a weak and diffuse superlattice (1/2 1/2 1/2) diffraction peak, indicating partially ordered domains (Randall & Bhalla, 1990 ▸; Burton *et al.*, 2005 ▸; Zhao *et al.*, 2009 ▸; Welberry *et al.*, 2006 ▸; Paściak *et al.*, 2013 ▸). Several models of cation ordering have been proposed for these materials (Davies *et al.*, 2008 ▸). The space–charge model was first put forward for PMN (Fanning *et al.*, 2000 ▸; Zhang *et al.*, 1996 ▸), in which the 1:1 ratio of Nb and Mg distribute alternately between the B′ and B′′ sites on the (111) planes, so that an unbalanced electrical charge exists in the checker-board-type ordered regions. Thus, the surrounding matrix must be Nb-rich in order to reach the overall 2:1 ratio given by the stoichiometry and electrical charge balance. This model was named α-COR in recent years (Kopecký *et al.*, 2016 ▸). But later, TEM and neutron diffraction on Zr/La-doped Pb(Mg_1/3_Ta_2/3_)O_3_ (PMT) samples, as well as atomic resolution *Z*-contrast imaging on PMN (Akbas & Davies, 1997 ▸, 2000 ▸, 2001 ▸; Dmowski *et al.*, 2002 ▸; Cantoni *et al.*, 2004 ▸; Montgomery *et al.*, 1999 ▸; Yan *et al.*, 1998 ▸; Jin *et al.*, 2001 ▸), showed that in the ordered region Ta^5+^(Nb^5+^) occupied the B′′ site, while Mg^2+^ and Ta^5+^(Nb^5+^) occupied the B′ site randomly with a 2:1 ratio. It is called the charge-balanced random-site model, or β-COR. However, most of the results are only based on chemically doped materials and research on pure Pb(B′_1/3_B′′_2/3_)O_3_-type perovskites is rather limited.

Generally, Pb(Cd_1/3_Nb_2/3_)O_3_ (PCN) is considered a relaxor material with cubic symmetry (Tomashpol’skii & Venevtsev, 1965 ▸; Ichinose *et al.*, 1971 ▸; Bąk *et al.*, 2008 ▸; Kajtoch *et al.*, 2008 ▸; Kajtoch, 1999 ▸, 2009 ▸; Pastor *et al.*, 2014 ▸). In relaxors, the magnitude of maximum dielectric permittivity usually decreases while the temperature of maximum dielectric permittivity (*T*_max_) shifts towards higher values when the measuring frequency increases. In contrast, in PCN, both *T*_max_ and the maximum dielectric permittivity shift to lower values with an increase in frequency (Kajtoch, 1999 ▸, 2009 ▸). However, studies on its structures and dielectric properties are still largely lacking. This is partly caused by difficulties in the synthesis of a pure perovskite phase of PCN, which always comes with a secondary pyrochlore phase (Kajtoch, 1999 ▸, 2009 ▸; Pastor *et al.*, 2014 ▸). To the best of our knowledge, single crystals and ceramics of PCN with a pure perovskite phase have never been successfully synthesized to date.

In this work, PCN single crystals and ceramics were successfully synthesized as a pure perovskite phase. The {1/2 1/2 1/2} superlattice reflections were investigated based on single-crystal synchrotron X-ray experiments, laboratory-based high-resolution X-ray diffraction and selected-area electron diffraction (SAED) methods. The chemically ordered structure was analysed by atomic resolution energy-dispersive X-ray spectroscopy (EDS). Temperature-dependent dielectric properties and hysteresis loops were also studied. It has been found that large coherent chemically ordered regions, consistent with the β-COR model, exist in PCN crystals and ceramics; both have two dielectric anomaly peaks and exhibit slightly different relaxation processes.

## 2. Experimental   

The Pb(Cd_1/3_Nb_2/3_)O_3_ single crystals were grown by the top-cooled solution growth method. Raw materials PbO (>99%), CdO (>99.5%) and Nb_2_O_5_ (>99%) were weighed according to the stoichiometric composition of PCN. PbO was selected as the flux, and the flux to the charge molar ratio was 7:3. Firstly, the mixed powder was put into a platinum crucible and heated to 1120°C in a cylindrical furnace. It was then soaked at this temperature for 48 h to achieve a full melt. A platinum wire was attached to the upper surface of the high-temperature solution to induce nucleation near the platinum wire. Then the temperature was gradually cooled to 800°C at a rate of 5°C h^−1^ to allow single crystals to grow. The system was then cooled to room temperature at a faster speed of 120°C h^−1^ to avoid the formation of other phases. Finally, the crystals were washed by immersing the solidified flux in 30% HNO_3_ solution. The as-grown single crystals exhibited an irregular shape and yellow colour. No inclusion or internal strain was observed under an optical microscope.

Pb(Cd_1/3_Nb_2/3_)O_3_ ceramics were prepared using a two-step solid-state reaction method and sintering process. First, the raw materials CdO (>99.5%) and Nb_2_O_5_ (>99%) were carefully ball milled in alcohol for 12 h and then calcined at 1100°C for 6 h to synthesize the precursor CdNb_2_O_6_. The calcined powder was then confirmed to be the columbite phase by X-ray diffraction (XRD). Next, the precursor CdNb_2_O_6_ and PbO (>99%) powders were mixed with a 4 wt% excess of PbO to compensate for PbO volatilization at high temperatures and then ball milled in alcohol for 12 h. The dried powders were calcined at 825°C for 4 h to obtain the perovskite phase and then reground in alcohol for 12 h. After calcination, the powders were granulated with a 5 wt% polyvinyl alcohol (PVA) binder and then pressed into pellets under a uniaxial pressure of 250 MPa with a diameter of 8 mm. The pellets were heated at 550°C for 0.5 h to evaporate the PVA binder and then sintered at 1025°C for 4 h. To minimize the vaporization of PbO, the pellets were buried in powders with the same composition. In addition, some PbZrO_3_ + 5% PbO powder was placed in the crucible to provide a saturated atmosphere of lead oxide.

The ceramics and some small crystals were ground into powder for phase and structure determination by X-ray powder diffraction using a PANalytical Empyrean diffractometer with a Cu *K*α_1_ X-ray tube. The scanning step size was set to 0.0065° with a 2θ range from 10 to 120°. The collected XRD data were then analysed by Rietveld refinement (Rietveld, 1969 ▸) using *TOPAS* Academic software (Coelho, 2005 ▸). Some pieces of crystals were polished parallel to the {110} planes with a thickness of 100 µm for optical observation. Polarized light microscopy experiments were performed with an Olympus BX-51 polarizing microscope equipped with a Linkam (THMS600E) heating stage. The PCN ceramics and crystal samples were coated with silver electrodes and fired at 500°C for 30 min for electrical measurements. The dielectric properties as a function of temperature were measured from room temperature to 500°C by using a precision impedance analyser (Agilent 4980A precise LCR meter) and a broad frequency dielectric spectrometer (NOVOCONTROL GmbH) from liquid-nitro­gen temperature up to 300°C at various frequencies. The hysteresis loops were displayed using a standardized ferroelectric test system (TF Analyzer 2000 Systems, Aix ACCT, Germany).

A piece of PCN crystal was selected and polished into an 80 × 80 × 500 µm needle for synchrotron experiments at the XRD1 beamline of Elettra Sincrotrone, Trieste, Italy. The data were collected using X-rays with an energy of 19 keV using a Pilatus 2M detctor. In the diffuse-scattering measurements the sample was rotated 360° around the ω axis with a Δω = 0.2° step and an exposure of 5 s, which allowed us to cover a full reciprocal-space sphere. An additional measurement with an exposure of 0.5 s and an aluminium attenuator was taken to avoid saturation of Bragg spots. Data were treated with the *SNBL* Toolbox (Dyadkin *et al.*, 2016 ▸), Bragg peaks were integrated with the program *CrysAlis*, while reciprocal-space images were reconstructed with *Xcavate* (Estermann & Steurer, 1998 ▸).

The PCN samples for Cs-TEM analysis were prepared by mechanical thinning from PCN ceramics. They were then Ar^+^ milled in a Gatan Precision Ion Polishing System. TEM investigations were performed on a JEM-ARM300F Atomic Resolution Electron Microscope equipped with double EDS detectors.

## 3. Results and discussion   

### 3.1. Long-range structure   

**Figure 1:**
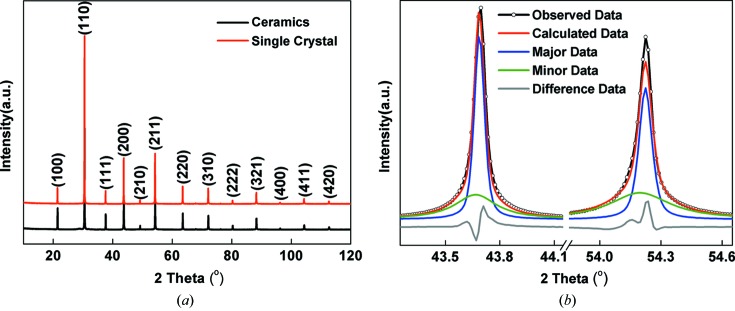
(*a*) X-ray diffraction patterns of the Pb(Cd_1/3_Nb_2/3_)O_3_ ceramics and single-crystal powder at room temperature; (*b*) {200} and {211} reflections showing the Rietveld refinement of the crushed single-crystal data using a cubic phase and a minor ‘diffuse’ phase.

The XRD patterns of PCN ceramics and powder crushed from single crystals at room temperature are shown in Fig. 1 ▸(*a*). All the Bragg peaks correspond to a perovskite cubic phase without any secondary pyrochlore phases. The diffraction data were Rietveld refined using a cubic 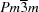 structure. The lattice parameter was calculated to be 4.1388 (9) Å, which is consistent with previous results (Kajtoch, 2009 ▸). However, underneath all the main Bragg reflections, there were diffuse intensities indicating possible local disorder. A good fit could only be achieved by adding a minor cubic phase just to model the diffuse intensities (Fig. 1 ▸ *b*). The single crystals have also been studied using polarized light microscopy in the temperature range −150 to 500°C. Extinction occurs in all directions in the whole temperature range, which indicates that the PCN crystal maintains long-range cubic symmetry without phase transitions in the temperature range studied.

### 3.2. Electrical properties   

**Figure 2:**
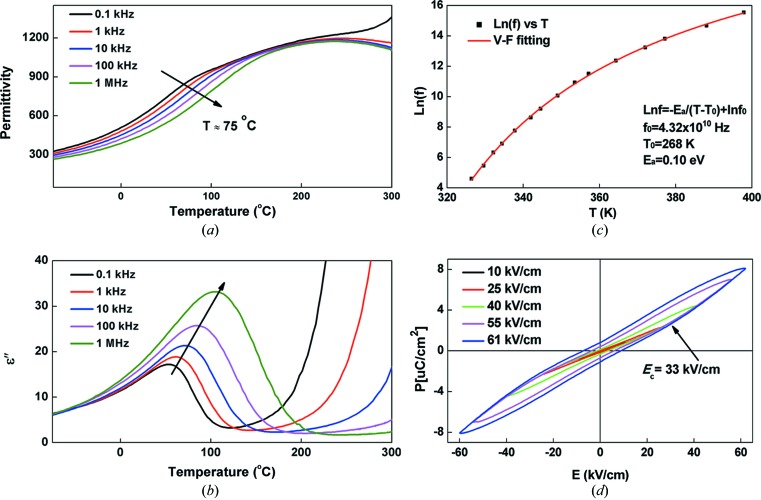
Electrical properties of single-crystal PCN: temperature dependences of the dielectric permittivity (*a*) real part and (*b*) imaginary part of a PCN single crystal at various frequencies. (*c*) Vogul–Fulcher fitting of the imaginary part of the dielectric permittivity. The squares are the experimental results and the red line is the fitted result. (*d*) P–E hysteresis loop of single-crystal PCN measured at room temperature with a frequency of 10 Hz.

Temperature dependences of the real and imaginary parts of dielectric permittivity have been measured in the temperature range −150 to 300°C and the frequency range 1 kHz to 1 MHz for single-crystal samples, as shown in Figs. 2 ▸(*a*) and 2(*b*). Generally, the PCN single-crystal samples showed a fairly broad diffuse dielectric peak around 270°C. The dielectric maximum temperature *T*_max_ is in good agreement with the literature (Kajtoch, 2009 ▸). An additional dielectric anomaly was observed at 75°C for the single-crystal sample. This anomaly was more pronounced at low frequencies and gradually became indistinguishable at high frequencies. Therefore, the dispersion can be regarded as a dielectric relaxation process. The frequency dependency is more clearly shown in the imaginary part of the dielectric permittivity ∊′′ plot (Fig. 2 ▸ *b*). Similar dielectric dispersions are also observed in the ceramic samples (see Fig. S1). Note that neither of the dielectric anomaly temperatures correspond to any average structural changes.

We performed Vogel–Fulcher (VF) law fitting, which is often utilized in relaxor ferroelectrics to describe the temperature and the frequency of dielectric permittivity peaks (Bokov & Ye, 2006 ▸), on the frequency dependence of the maximum temperature in ∊′′(*T*) peaks using the following equation
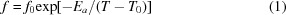
where *f* is the measurement frequency, *T* is the peak temperature in ∊′′(*T*), and *f*_0_, *E_a_* and *T*_0_ are the fitting parameters. The fitting result is shown in Fig. 2 ▸(*c*), demonstrating that the frequency dependence of the dielectric permittivity follows a Vogel–Fulcher-type relationship. The fitted parameters are physically reasonable with an *E_a_* of ∼0.1 eV, an *f*_0_ of ∼10^10^ Hz and a Vogel–Fulcher temperature of ∼268 K. However, the temperature range where the frequency dependence occurs does not coincide with *T*_max_. The possible reason is that the relaxation process in PCN is not the relaxation of polar nano-regions like in PMN. Also, this dielectric anomaly is found to be in a different temperature range in the ceramics samples (Fig. S1).

Fig. 2 ▸(*d*) shows the *P*–*E* hysteresis loop for a piece of PCN single crystal. Under high electric field (above 33 kV cm^−1^), it is possible to induce a polar state with remnant polarization. Ceramic samples show very similar hysteresis loops (Fig. S2). Overall, the electrical properties of both the PCN single crystals and ceramics display a relaxor-like behaviour and local polarizations. On the other hand, to understand whether the local polarizations form polar regions or a glass-like state requires more detailed local structural studies.

### 3.3. X-ray diffraction and diffuse-scattering evidence for chemical ordering and displacive disorder   

The analysis of diffuse scattering (DS) signals has proven to be a powerful tool to probe the local structural information in a crystallized material. In total X-ray scattering, the Bragg diffraction reveals long-range ordering in the crystal while diffuse scattering arises from any kind of departures from the ideal units correlated at short range (Welberry, 2004 ▸). In recent years, many experiments studying diffuse scattering intensities in Pb-based relaxors have been performed (Vakhrushev *et al.*, 1996 ▸, 2005 ▸; Naberezhnov *et al.*, 1999 ▸; Hirota *et al.*, 2002 ▸; Xu *et al.*, 2004 ▸; Welberry *et al.*, 2005 ▸; Paściak *et al.*, 2012 ▸, 2013 ▸; Bosak *et al.*, 2012 ▸), which helped to reveal details concerning the correlations between atoms over a scale of a few nanometres.

**Figure 3:**
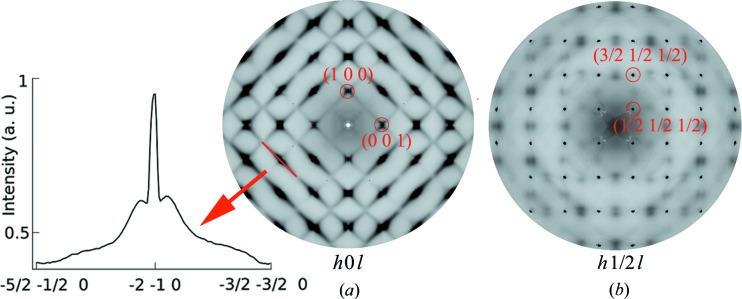
Diffuse scattering patterns of a PCN single crystal at room temperature at the (*a*) *h*0*l* and (*b*) *h* 1/2 *l* layers. Cubic symmetry has been applied and the intensity is displayed in a logarithmic scale. The inset of (*a*) shows the one-dimensional intensity profile through the −2−10 reflection.

The diffuse scattering intensities from the *h*0*l* main Bragg layers are shown in Fig. 3 ▸(*a*). The distribution of intensities is qualitatively similar to that found in other Pb-based relaxors with the characteristic ellipsoidal and butterfly-like shapes around the Bragg reflections extending into diffuse streaks running along the 〈110〉 directions. This indicates that there are local atomic displacements away from the cubic position in the PCN crystals that are similar to other Pb-based relaxors. What makes PCN different is the fact that the maximum values of the DS distribution around the Bragg spots with *h* + *k* + *l* = odd indices are slightly off the nodes of the reciprocal space [visible in the intensity profile through the −2−10 spot, shown as the inset in Fig. 3 ▸(*a*)]. The vicinity of these Bragg spots is supposed to be populated mostly with DS signals related to optic-like displacements of atoms (Hirota *et al.*, 2002 ▸). Therefore, it is possible that the local polar structure cannot be characterized as nanoscale domains with homogeneous polarization (which would necessarily lead to DS with maxima exactly at the positions of the Bragg reflections) in PCN. Instead, short-range order can incorporate regions with dispersed distribution of polarizations with a tendency towards finite-wavelength modulation (Randall *et al.*, 1989 ▸). This picture would explain the sluggish behaviour of the permittivity and underline possible ferroelectric–antiferroelectric competition in PCN.

There are also intensities occurring on the superlattice *h* 1/2 *l* layer – in addition to blurred spots that emerge as a cross section with 〈110〉-oriented DS rods [some of which are visible in Fig. 3 ▸(*a*)], there are sharp superlattice diffraction spots, *e.g.* (1/2 1/2 1/2), (3/2 1/2 1/2) and (1/2 1/2 3/2) *etc.* (Fig. 3 ▸ *b*). They occupy all the half-integer reflections, with *h*, *k* and *l* equal and not equal to each other. In general, either the octahedral tilt or the chemical ordering structure can be related to the existence of the half-integer reflections in the diffraction pattern of a perovskite crystal. But in the octa­hedral tilt case, all the half-integer reflections with *h* = *k* = *l* are in extinction (Glazer, 1975 ▸). Therefore, the half-integer scattering intensities observed in this experiment suggest B-site chemical ordering in PCN crystals. The full width at half-maximum of these spots is practically the same as for parent Bragg reflections, which means that within the resolution limit of the experiment, the spatial extent of this type of ordering is comparable with the coherence of the parent average structure.

**Figure 4:**
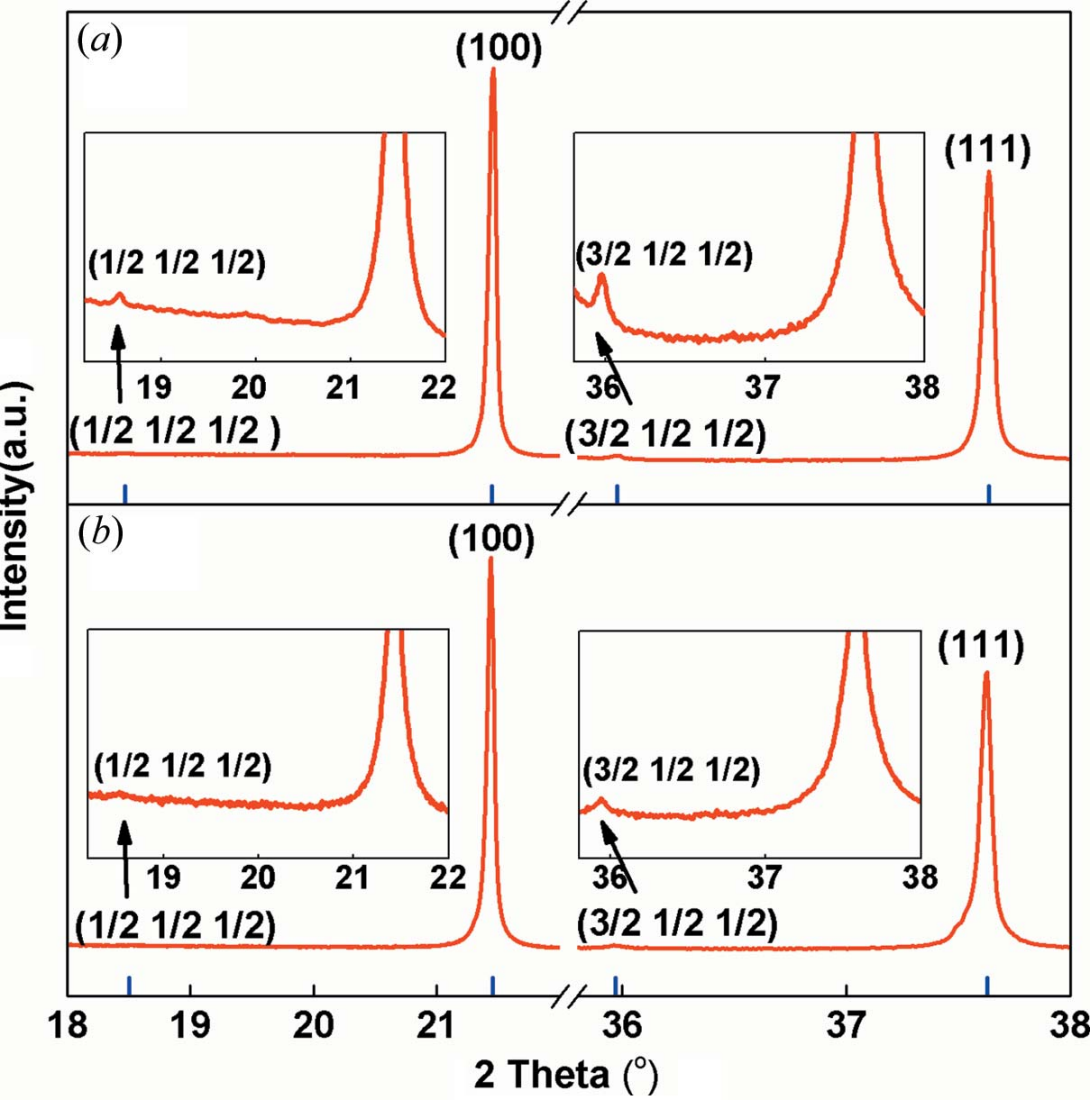
Superlattice reflections {1/2 1/2 1/2} and {3/2 1/2 1/2} at 2θ = 18.5° and 35.95° positions of (*a*) a PCN ceramic and (*b*) a PCN single crystal. The ordinate values of all inset images are given on a logarithmic scale.

With this large chemically ordered correlation length, it may be possible to observe and analyse the characteristic {1/2 1/2 1/2} reflections in PCN single crystals and ceramics using a lab-based X-ray diffractometer. Because of their very low intensities, the scanning speed is set as slow as 3500 s per step to achieve a high flux. The obtained diffraction data for the PCN ceramics are shown in Figs. 4 ▸(*a*) and 4(*b*). Two superlattice reflections {1/2 1/2 1/2} and {3/2 1/2 1/2} with weak intensities can be clearly observed at the 2θ = 18.5 and 35.95° positions. Compared with PMN, the detectable superlattice diffraction peaks in PCN again indicate that the size of CORs in PCN is relatively large. While the CORs in PMN only have a size of 2–5 nm (Mathan *et al.*, 1991 ▸), the superlattice reflections have never been detected by conventional X-ray diffraction methods.

### 3.4. Ionic distribution   

**Figure 5:**
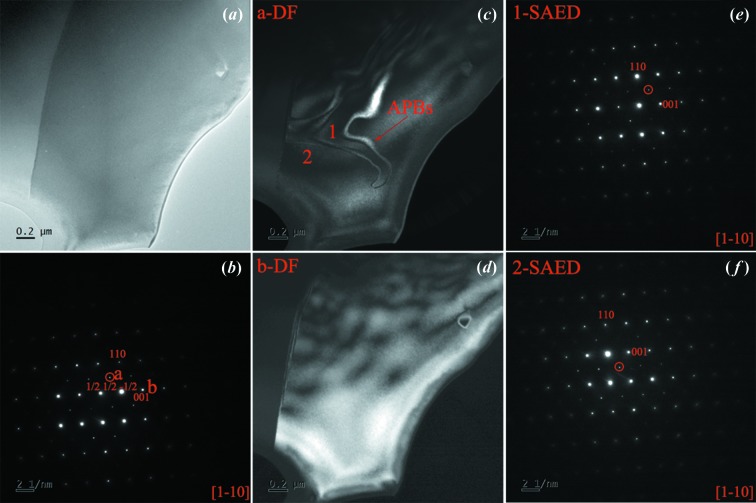
(*a*) TEM image of PCN. (*b*) SAED pattern with the [1−10] zone axis showing the {1/2 1/2 1/2} superlattice reflection. (*c*) DF image of PCN using the reflection (1/2 1/2 −1/2). (*d*) DF image using the reflection (001). (*e*)–(*f*) The SAED patterns of (*e*) the region marked 1 and (*f*) the region marked 2 in image (*c*), viewed along the [1–10] zone axis.

In order to directly observe the B-site chemical ordering in PCN, TEM was used. A TEM image of a PCN ceramic sample is shown in Fig. 5 ▸(*a*). The SAED pattern of Fig. 5 ▸(*a*) is displayed in Fig. 5 ▸(*b*), viewed along the [1–10] zone axis. Similar to the diffuse scattering results, the superlattice {1/2 1/2 1/2} reflections are clearly seen in the whole grain regions, and these half-integer reflections correspond to the doubled unit cell caused by the B-site ordering. The dark-field (DF) images were acquired using the (1/2 1/2 −1/2) and (001) reflections, as shown in Figs. 5 ▸(*c*) and 5 ▸(*d*), respectively. It is found that the DF image shows a homogeneous contrast in the whole grain in Fig. 5 ▸(*c*), which suggests that ordered regions are distributed evenly in the whole PCN sample. Antiphase domain boundaries (APBs) are also distinguished by wavy lines in Fig. 5 ▸(*c*). Two regions on both sides of the antiphase domain boundaries were selected for observing the distribution of ordered structures by SAED. The SAED patterns with the [1–10] zone axis marked 1 and 2 are shown in Figs. 5 ▸(*e*) and 5(*f*), respectively. Superlattice diffraction {1/2 1/2 1/2} exists on both sides of the APBs. Thus, PCN has a large coherent chemical ordering in all regions.

**Figure 6:**
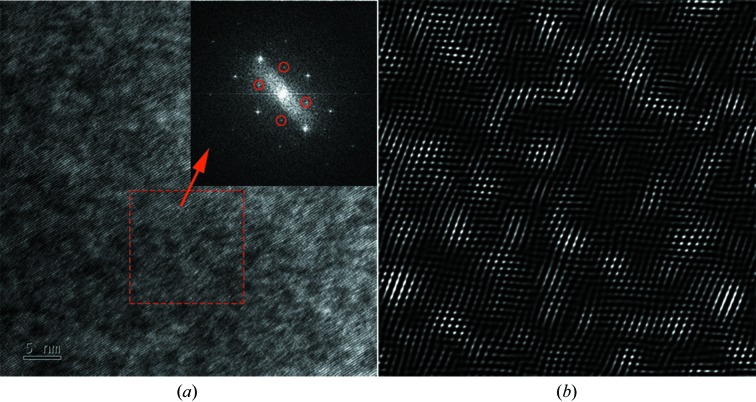
(*a*) High-resolution electron microscopy image of PCN and the fast Fourier transform patterns of the marked region as an inset. (*b*) The inverse Fourier transform of the inset image in (*a*) where only the superlattice reflections are included.

Further information on the chemical ordering of the PCN structure can be accessed with high-resolution transmission electron microscopy (HRTEM) experiments. The HRTEM image of the PCN sample is shown in Fig. 6 ▸(*a*), which is also observed along the [1–10] zone axis. The square regions in Fig. 6 ▸(*a*) are selected to be Fourier transformed for reviewing the diffraction pattern. The Fourier transform pattern is presented as an inset in Fig. 6 ▸(*a*). Some weak superlattice diffraction peaks are also observed and marked by circles. Fig. 6 ▸(*b*) shows the inverse Fourier transform patterns of the inset image in Fig. 6 ▸(*a*), where only the superlattice reflections are selected, changing the information displayed from reciprocal space to real space. The dark and bright regions are distributed randomly, but all areas are ordered. Only the relative intensities are different. In the relatively bright area, the lattice may have smaller distortion, while the distortion is larger in the relatively dark area. This is also the reason why the antiphase domain boundaries often appear between the bright and dark areas.

**Figure 7:**
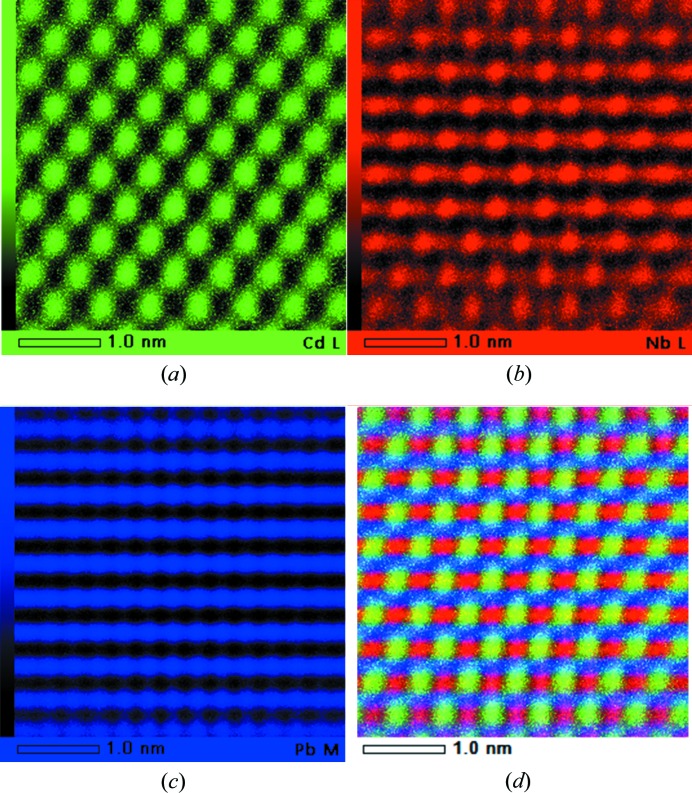
The atomic resolution energy-dispersive X-ray spectroscopy (EDS) mapping of (*a*) Cd, (*b*) Nb and (*c*) Pb. (*d*) Overlapping maps of the three elements above. Note that in (*d*), the Cd positions show a lime colour, which is a mixture of green (Cd) and red (Nb) colours in (*a*) and (*b*). All the maps are viewed along [110].

In order to investigate what kind of B-site chemical ordering exists in PCN, we also applied the atomic resolution EDS method. In the case of perovskites, if the B cations align in a checker-board arrangement, different types of atomic columns can be viewed along the [110] zone axis. EDS mappings taken along the [110] zone axis of the Cd, Nb and Pb ions are displayed in Figs. 7 ▸(*a*)–7(*c*), respectively. It is observed that the Cd ions only occupy every second nearest neighbour of the B sites (B′ positions), as seen in Fig. 7 ▸(*a*). In Fig. 7 ▸(*b*), it can be clearly resolved that the Nb ions occupy the B′′ positions where there are no Cd ions. However, there are also signals for Nb ions in the B′ positions, indicating that some Nb ions occupy the B′ position. The weak signal is caused by the fact that the B′ atom column consists of both Cd and Nb ions. Moreover, the fluctuation of the Cd and Nb signals in the B′ positions suggests that they are distributed randomly over the B′ positions. This is also confirmed by the overlapping maps of three atoms in Fig. 7 ▸(*d*). From the DF experiment, we know that PCN is completely ordered in whole regions. Combined with the B-site ionic distribution from the atomic resolved EDS mapping, it is concluded that the B-site ordering model obtained for PCN is consistent with the fully ordered β-COR model. Namely, the Nb ions occupy every second nearest-neighbour B site (B′′), while the Cd and Nb ions are distributed randomly over the B′ sites in a 2:1 ratio. Thus, the formula of the chemically ordered regions can be maintained as Pb[(Cd_2/3_Nb_1/3_)_1/2_Nb_1/2_]O_3_.

With a clear image of the chemically ordered structure determined, we revisited the single-crystal X-ray diffraction data and performed quantitative analysis. The procedure has been used in many previous works (*e.g.* Perrin *et al.*, 2001 ▸) in which a ratio of selected superlattice and parent-lattice peaks is compared between the experimental and calculated data from an ordered structural model. We note that (3/2 1/2 1/2) is stronger than (1/2 1/2 1/2) and so it is convenient to define an order parameter as 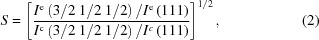
where *I* ^e^ and *I* ^c^ are the experimental and calculated intensities, respectively. As a model, we consider the β-COR 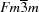 structure, in which one site (B′′) of the checker-board-like B-site sublattice is occupied exclusively by Nb and the other (B′) randomly by Cd and Nb in a 2:1 ratio. This structure has only one free parameter (besides the lattice constant *a*, which we set according to the experimental value as 8.28 Å) – a special position of the oxygen atom that defines two different sizes of octahedra for different B sites. If we leave the oxygen atom exactly in between B′ and B′′, the value of *S* for the single-crystal diffraction data (taking the average of all equivalent reflections in the experimental data set) is 1.23. This means that the integrated intensities of superlattice spots (3/2 1/2 1/2) (for simplicity we keep the indexing of the parent 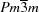 single perovskite unit cell) are substantially higher than the value that the completely ordered structural model predicts. However, we note that Nb and Cd have very distinct ionic radii (0.64 *versus* 0.95 Å) and it is reasonable to expect that the oxygen network adapts to this difference. In PSN for example, where the disparity between Sc and Nb is still smaller, first-principles calculations have indeed shown that there are two different sizes of octahedra expected for an ordered structure (Paściak *et al.*, 2016 ▸). When we modify the oxygen position in our 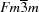 model such that the Nb—O and Cd_2/3_Nb_1/3_—O distances are proportional to their respective ionic radii (*d*_Nb−O_ = 1.97 Å, 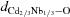 = 2.17 Å), the 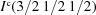 intensity is enhanced and the order parameter changes to *S* = 1.06. Remaining ‘excessive order’ could be related to the intensities of parent peaks being affected by Pb disorder as well as possible tilting of the octahedra that would further increase the intensities of the superlattice spots (3/2 1/2 1/2). Nevertheless, this result, together with the sharpness of the superlattice spots, suggests that PCN exhibits long-range chemical order that is effectively present in the whole body of the sample.

We have also performed Rietveld refinement using the fully β-ordered chemical arrangement against the powder diffraction data. Similar to the strategy described in Section 3.1, a minor cubic phase needed to be included to simulate the diffuse scattering intensities. By having a major 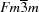 structure with one site (B′′) occupied only by Nb and the other (B′) randomly by Cd and Nb in a 2:1 ratio, a good agreement is achieved between the observed and calculated data, including the area with the superlattice {3/2 1/2 1/2} peak. The fitted result in the 2θ region containing both {3/2 1/2 1/2} and {111} reflections is shown in Fig. S3 of the supporting information. A CIF containing the refined structural information is provided with this paper.

This further confirms that the relative ionic radii of the B-site cations play an important role in the degree of ordering: the larger the size difference between B′ and B′′ ions, the higher the degree of cation ordering (Setter & Cross, 1980*a* ▸; Chen *et al.*, 1989 ▸). In the common Pb-based relaxors, the ionic radii are *R*_Ni_ (0.69 Å) < *R*_Mg_ (0.72 Å) < *R*_Zn_ (0.74 Å) < *R*_Cd_ (0.95 Å), and so the degrees of chemical ordering are possibly Pb(Ni_1/3_Nb_2/3_)O_3_ (PNN) < PMN < PZN < PCN. It has been reported that there is almost no diffraction peak of this superlattice in PNN (Sharma *et al.*, 1993 ▸). The chemically ordered region sizes in PMN are found to be larger than those in PNN (Dmowski *et al.*, 2002 ▸). The superlattice diffraction spots in PZN are broad and of small intensity, which is similar to PMN (Paściak *et al.*, 2013 ▸; Kopecký *et al.*, 2016 ▸). It is shown from this work that PMN and PZN exhibit a lower degree of chemical ordering than PCN, which has a fully ordered chemical distribution.

## 4. Conclusions   

PCN single crystals and ceramics of pure perovskite phase have been successfully prepared by the top-cooled solution growth method and the two-step solid-state reaction method, respectively. PCN maintains a cubic average structure in the temperature range −150 to 500°C. A long-range chemically ordered structure is confirmed by a variety of experiments. Atomic resolution EDS mapping reveals that it is β-COR type. We believe that this structural feature is related to the possible relaxor nature of PCN and similar types of complex perovskites. The significant ionic radius difference between B′ and B′′ makes the CORs in PCN large enough to be observed and analysed, while the same type of chemical correlations in PMN only exist on a smaller scale. On the other hand, although cation displacive disorder is indicated by the diffuse scattering intensity, the distribution of the local polarizations in PCN is found to be different from PMN and PZN. This may cause the difference in the frequency dependence of dielectric permittivity between PCN and other relaxors. Therefore, our direct observations on the chemical arrangements in PCN combined with the dielectric permittivity experiments potentially open up an opportunity to systematically study and understand the structural origin of the relaxor behaviour from a partial chemical ordering perspective.

## Supplementary Material

Crystal structure: contains datablock(s) PCN_publ, PCN_overall, PCN_phase_1, PCN_phase_2. DOI: 10.1107/S2052252518013805/lt5010sup1.cif

Additional figures. DOI: 10.1107/S2052252518013805/lt5010sup2.pdf

CCDC references: 1871645, 1871646
