# Supplementary material for: Determination of chemical ordering in the complex perovskite Pb(Cd1/3Nb2/3)O3
Source: IUCrJ. 2018 Oct 24;5(Pt 6):808–15. doi: 10.1107/S2052252518013805 (PMC6211540; doi:10.1107/S2052252518013805)
Supplement: Supplementary file 2 [file m-05-00808-sup2.pdf]

IUCrJ

Volume 6 (2019)

Supporting information for article:

Determination of chemical ordering in the complex perovskite Pb(Cd1/3Nb2/3)O3

Caiyan Wang, Zhengqian Fu, Nan Zhang, Marek Paściak, Jian Zhuang, Zenghui Liu, Wei Ren and Zuo-guang Ye

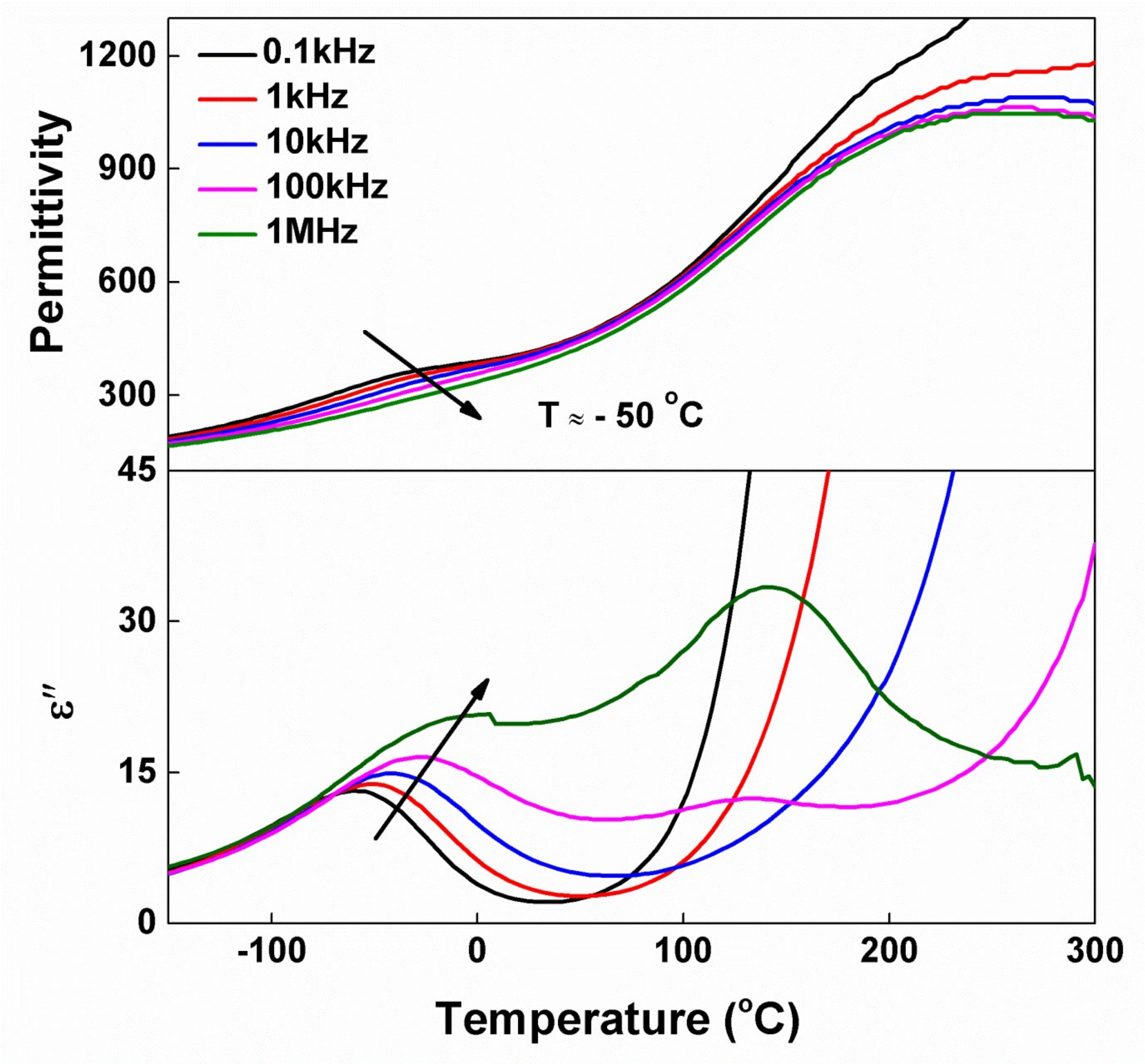

1 Temperature dependence of the real and imaginary part of dielectric permittivity of PCN ceramics with various frequencies.

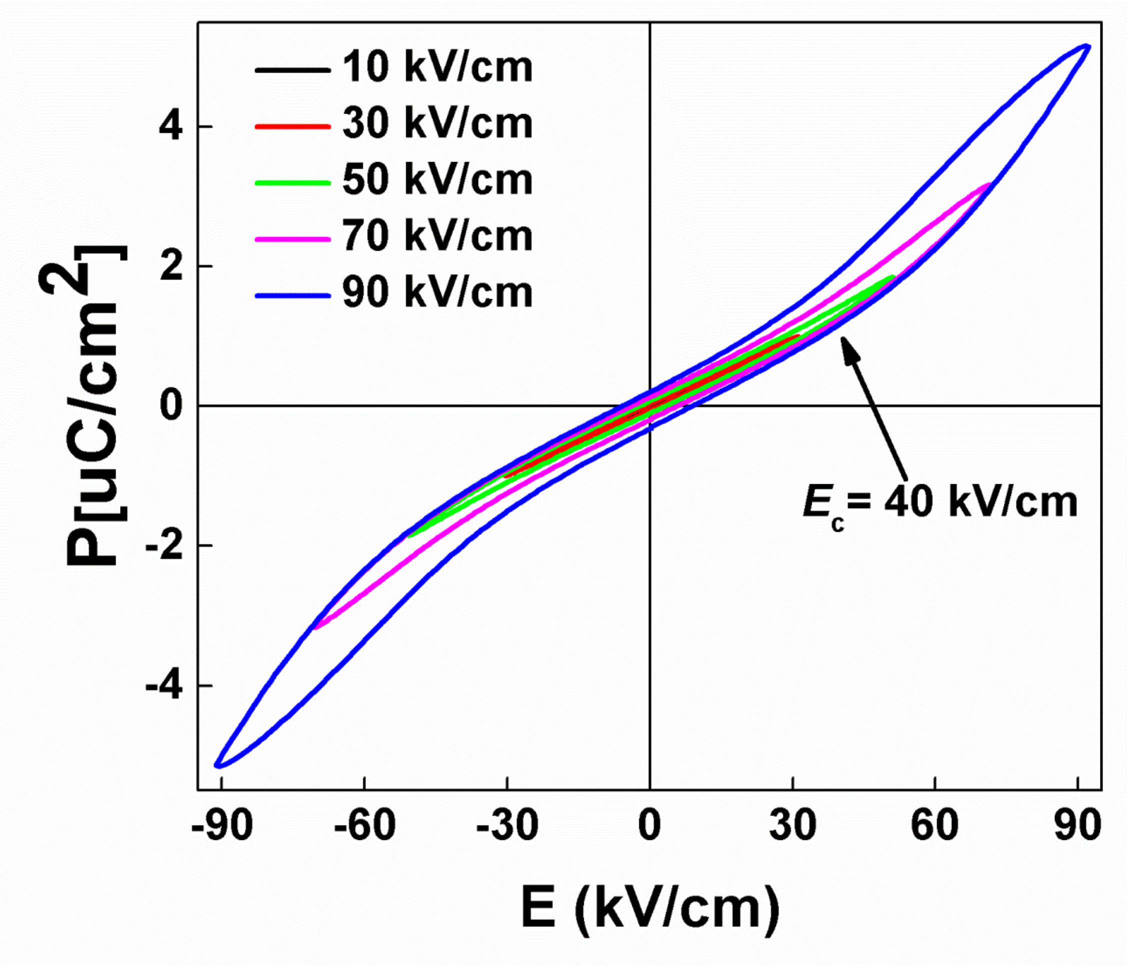

2 Hysteresis loops of PCN ceramics samples with the various electric fields at room temperature with a frequency of 10 Hz.

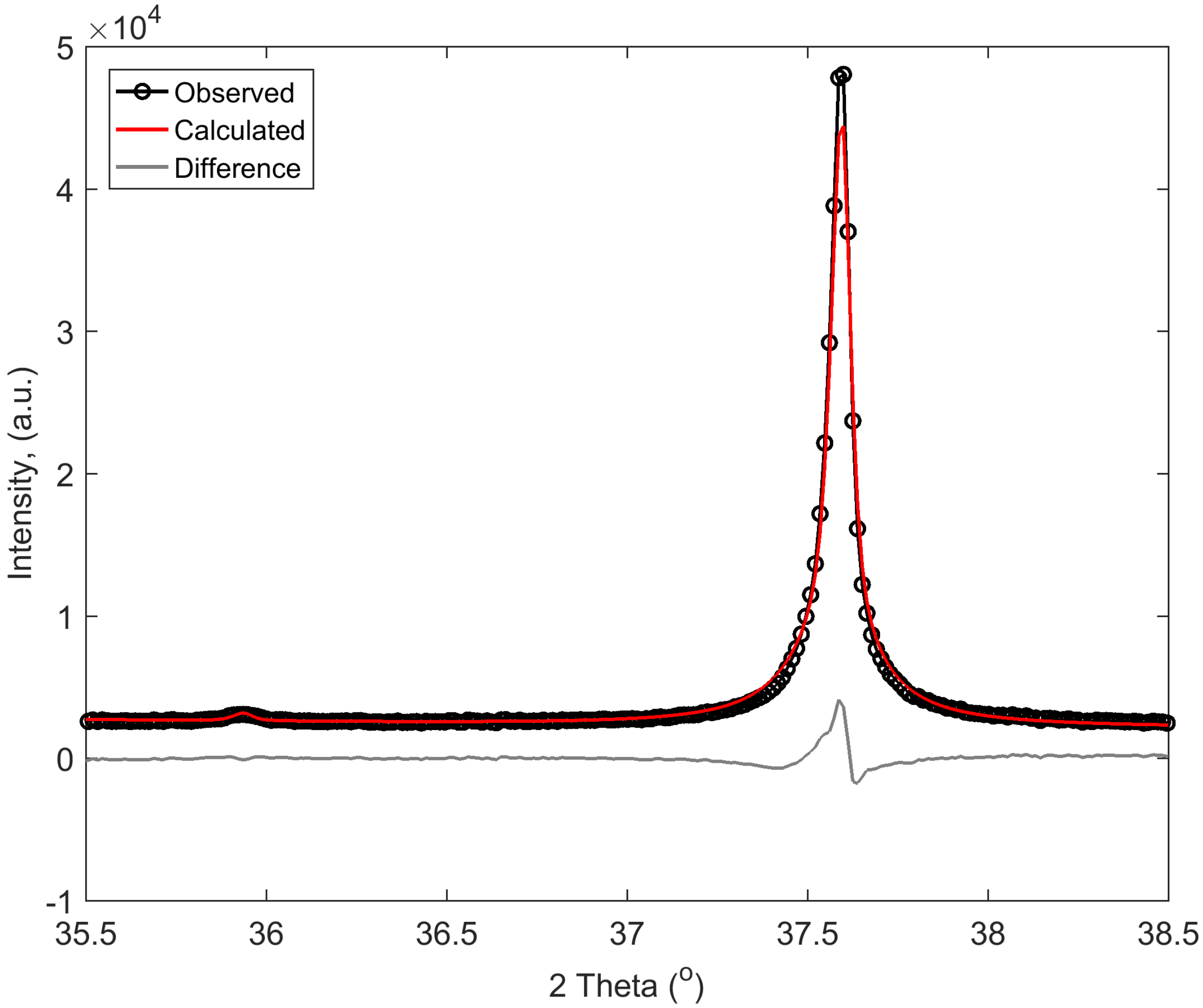

3 Rietveld refined profiles for the 2θ range showing the fitting result of {3/2 1/2 1/2} and {1 1 1} reflections.
